# 3-Ethyl-6-(4-fluoro­phen­yl)-7*H*-1,2,4-triazolo[3,4-*b*][1,3,4]thia­diazine

**DOI:** 10.1107/S160053681202185X

**Published:** 2012-05-23

**Authors:** S. Jeyaseelan, H. C. Devarajegowda, R. Sathishkumar, Agnes Sylvia D’souza, Alphonsus D’souza

**Affiliations:** a Department of Physics, Yuvaraja’s College (Constituent College), University of Mysore, Mysore 570 005, Karnataka, India; b Solid State and Structural Chemistry Unit, Indian Institute of Science, Bangalore 12, Karnataka, India; c Department of Physics, St Philomena’s College (Autonomous), Mysore 570 015, Karnataka, India

## Abstract

In the title compound, C_12_H_11_FN_4_S, the thia­diazine ring adopts a twist-boat conformation. The dihedral angle between the triazolothia­diazine system and the benzene ring is 10.54 (9)°. The crystal structure is characterized by C—H⋯N hydrogen bonds. The crystal packing also exhibits π–π inter­actions, with a centroid–centroid distance of 3.6348 (15) Å.

## Related literature

For biological properties of triazolothia­diazines, see: Feng *et al.* (1992 ▶); Mohan & Anjaneyalu (1987 ▶); Holla *et al.* (2001 ▶); Walser *et al.* (1991 ▶); Hirota *et al.* (1991 ▶); Bradbury & Rivett (1991 ▶); Heindel & Reid (1980 ▶); Heidelberger *et al.* (1957 ▶). For related structures, see: Andersson & MacGowan (2003 ▶); Novak *et al.* (2006 ▶).
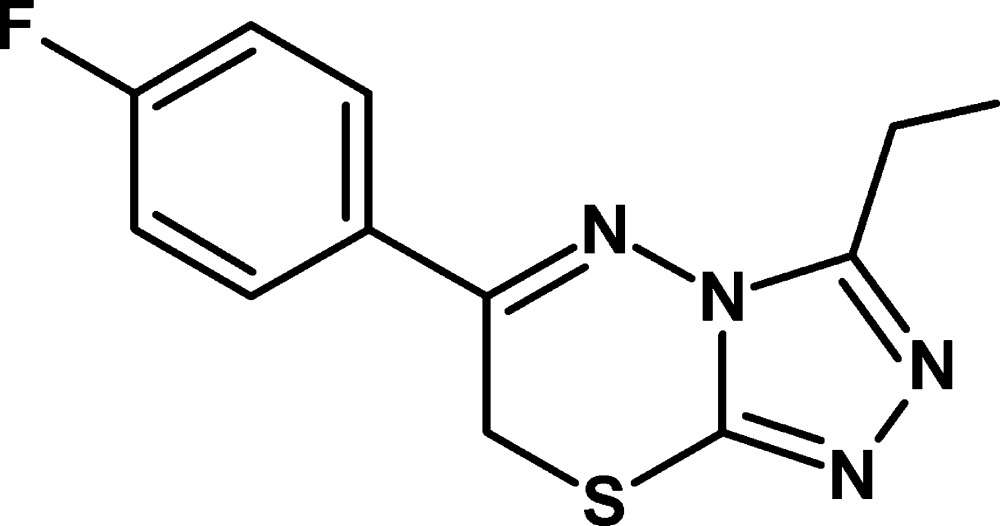


## Experimental

### 

#### Crystal data

C_12_H_11_FN_4_S
*M* *_r_* = 262.31
Monoclinic, 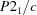

*a* = 13.322 (3) Å
*b* = 13.017 (3) Å
*c* = 7.1912 (16) Å
β = 105.308 (4)°
*V* = 1202.8 (4) Å^3^
*Z* = 4
Mo *K*α radiation
μ = 0.27 mm^−1^
*T* = 293 K
0.24 × 0.20 × 0.12 mm

#### Data collection

Bruker SMART CCD area-detector diffractometer
Absorption correction: multi-scan (*SADABS*; Bruker, 2001 ▶) *T*_min_ = 0.770, *T*_max_ = 1.000
11097 measured reflections
2119 independent reflections
1828 reflections with *I* > 2σ(*I*)
*R*_int_ = 0.023

#### Refinement

*R*[*F* ^2^ > 2σ(*F* ^2^)] = 0.041
*wR*(*F* ^2^) = 0.106
*S* = 1.06
2119 reflections
164 parameters
H-atom parameters constrained
Δρ_max_ = 0.22 e Å^−3^
Δρ_min_ = −0.22 e Å^−3^

### 

Data collection: *SMART* (Bruker, 2001 ▶); cell refinement: *SAINT* (Bruker, 2001 ▶); data reduction: *SAINT*; program(s) used to solve structure: *SHELXS97* (Sheldrick, 2008 ▶); program(s) used to refine structure: *SHELXL97* (Sheldrick, 2008 ▶); molecular graphics: *ORTEP-3* (Farrugia, 1997 ▶); software used to prepare material for publication: *SHELXL97*.

## Supplementary Material

Crystal structure: contains datablock(s) I, global. DOI: 10.1107/S160053681202185X/bt5922sup1.cif

Structure factors: contains datablock(s) I. DOI: 10.1107/S160053681202185X/bt5922Isup2.hkl

Supplementary material file. DOI: 10.1107/S160053681202185X/bt5922Isup3.cml

Additional supplementary materials:  crystallographic information; 3D view; checkCIF report

## Figures and Tables

**Table 1:** Hydrogen-bond geometry (Å, °)

*D*—H⋯*A*	*D*—H	H⋯*A*	*D*⋯*A*	*D*—H⋯*A*
C1—H1⋯N2^i^	0.93	2.51	3.428 (3)	172
C8—H8*A*⋯N1^ii^	0.97	2.50	3.410 (3)	156
C8—H8*B*⋯N2^i^	0.97	2.30	3.228 (3)	160

Symmetry codes: (i) 
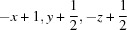
; (ii) 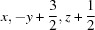.

**Fig. 1.**
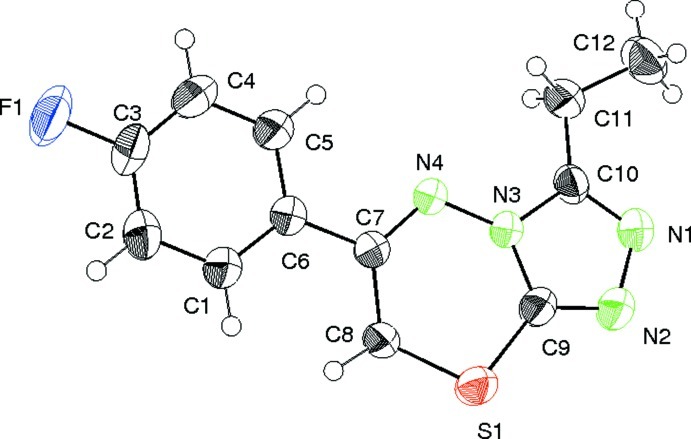
The molecular structure of the title compound. Displacement ellipsoids are drawn at the 50% probability level.

**Fig. 2.**
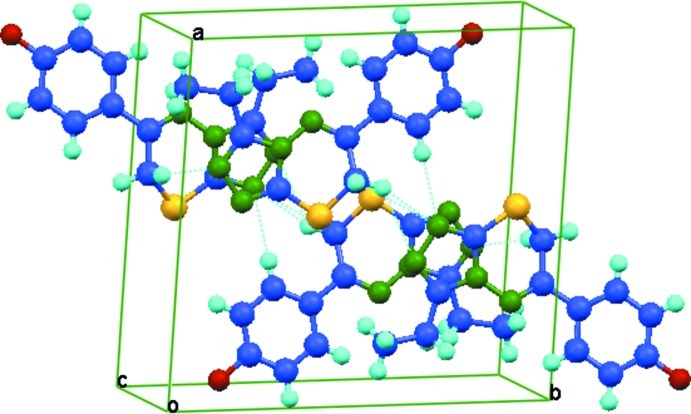
The packing of molecules in the title structure.

## supplementary crystallographic information

### Comment

Triazoles fused with six membered ring systems are found to be associated with diverse pharmacological activity. A number of thiadiazines have been shown to exhibit antimicrobial (Feng *et al.*, 1992) and dieretic properties (Mohan *et al.*, 1987) and also serves as photographic couplers (Holla *et al.*, 2001). The analgesic, anti-asthmatic, diuretic, anti-hypertensive, anti-cholinergic, antibacterial, antifungal, anti-inflammatory, hypoglycemic, anti-tubercular and antiviral properties exhibited by various N-bridged heterocycles derived from a variety of 4-amino-5-mercapto-1,2,4-triazoles, have made them an important chemotherapeutic agents (Walser *et al.*, 1991; Hirota *et al.*, 1991; Bradbury *et al.*, 1991). The 1,2,4- triazoles nucleus has recently been incorporated into a wide variety of therapeutically interesting drugs including H1/H2 histamine receptor blockers, cholinesterase active agents, *CNS* stimulants, anti-anxiety agents and sedatives (Heindel *et al.*, 1980). Further fluorinated heterocycles have been shown to possess wide variety of biocidal activities. Compounds such as fluorouracil and fluoroquinolone have been used as anticancer agents and antibiotics (Heidelberger *et al.*, 1957; Andersson *et al.*, 2003; Novak *et al.*, 2006).

The asymmetric unit of 3-ethyl-6-(4-fluorophenyl)-7*H*-[1,2,4]triazolo[3,4-*b*] [1,3,4]thiadiazine is shown in Fig. 1. The triazolo-thiadiazine ring system is not planar. The dihedral angle between the triazolo-thiadiazine ring system (S1/N1–N4/C7–C10) and the benzene ring (C1–C6) is 10.54 (9)°.

In the crystal structure (Fig. 2), the molecules are connected *via* intermolecular C1—H1···N2, C8—H8A···N1 and C8—H8B···N2 hydrogen bonds (Table 1). Furthermore, the crystal structure features a π-π interaction, with a centroid-centroid *Cg*1 (C9/C10/N1–N3) distance of 3.5728 (16) Å.

### Experimental

A mixture of triazole (1) (0.01 mol) and *p*-fluorophenacyl bromide (0.01 mol) in ethanol (25 ml) was heated under reflux for 1–2 hrs. The reaction mixture was cooled to room temparature and neutralized with sodium acetate (5%). The precipitated triazolothiadiazines were collected by filtration, washed with water and recrystallized from ethanol. Yield 82%; m.p.455 K.

### Refinement

All H atoms were positioned geometrically, with C—H = 0.93 Å for aromatic H, C—H = 0.97 Å for methylene H and C—H = 0.96 Å for methyl H, and refined using a riding model with *U*_iso_(H) = 1.5*U*_eq_(C) for methyl H and *U*_iso_(H) = 1.2*U*_eq_(C) for all other H.

### Crystal data

**Table:**

C_12_H_11_FN_4_S	*F*(000) = 544
*M**_r_* = 262.31	*D*_x_ = 1.449 Mg m^−^^3^
Monoclinic, *P*2_1_/*c*	Melting point: 455 K
Hall symbol: -P 2ybc	Mo *K*α radiation, λ = 0.71073 Å
*a* = 13.322 (3) Å	Cell parameters from 2119 reflections
*b* = 13.017 (3) Å	θ = 2.2–25.0°
*c* = 7.1912 (16) Å	µ = 0.27 mm^−^^1^
β = 105.308 (4)°	*T* = 293 K
*V* = 1202.8 (4) Å^3^	Plate, colourless
*Z* = 4	0.24 × 0.20 × 0.12 mm

### Data collection

**Table:**

Bruker SMART CCD area-detector diffractometer	2119 independent reflections
Radiation source: fine-focus sealed tube	1828 reflections with *I* > 2σ(*I*)
Graphite monochromator	*R*_int_ = 0.023
ω and φ scans	θ_max_ = 25.0°, θ_min_ = 2.2°
Absorption correction: multi-scan (*SADABS*; Bruker, 2001)	*h* = −15→15
*T*_min_ = 0.770, *T*_max_ = 1.000	*k* = −15→15
11097 measured reflections	*l* = −8→8

### Refinement

**Table:**

Refinement on *F*^2^	Primary atom site location: structure-invariant direct methods
Least-squares matrix: full	Secondary atom site location: difference Fourier map
*R*[*F*^2^ > 2σ(*F*^2^)] = 0.041	Hydrogen site location: inferred from neighbouring sites
*wR*(*F*^2^) = 0.106	H-atom parameters constrained
*S* = 1.06	*w* = 1/[σ^2^(*F*_o_^2^) + (0.0487*P*)^2^ + 0.4632*P*] where *P* = (*F*_o_^2^ + 2*F*_c_^2^)/3
2119 reflections	(Δ/σ)_max_ < 0.001
164 parameters	Δρ_max_ = 0.22 e Å^−^^3^
0 restraints	Δρ_min_ = −0.22 e Å^−^^3^

### Special details

**Table:**

Geometry. All s.u.'s (except the s.u. in the dihedral angle between two l.s. planes) are estimated using the full covariance matrix. The cell s.u.'s are taken into account individually in the estimation of s.u.'s in distances, angles and torsion angles; correlations between s.u.'s in cell parameters are only used when they are defined by crystal symmetry. An approximate (isotropic) treatment of cell s.u.'s is used for estimating s.u.'s involving l.s. planes.
Refinement. Refinement of *F*^2^ against ALL reflections. The weighted *R*-factor *wR* and goodness of fit *S* are based on *F*^2^, conventional *R*-factors *R* are based on *F*, with *F* set to zero for negative *F*^2^. The threshold expression of *F*^2^ > 2σ(*F*^2^) is used only for calculating *R*-factors(gt) *etc*. and is not relevant to the choice of reflections for refinement. *R*-factors based on *F*^2^ are statistically about twice as large as those based on *F*, and *R*- factors based on ALL data will be even larger.

### Fractional atomic coordinates and isotropic or equivalent isotropic displacement parameters (Å^2^)

**Table:**

	*x*	*y*	*z*	*U*_iso_*/*U*_eq_	
S1	0.50854 (4)	0.90687 (4)	0.19250 (9)	0.0542 (2)	
F1	0.05432 (12)	1.31988 (11)	−0.1252 (2)	0.0845 (5)	
N1	0.37247 (14)	0.64191 (13)	0.1417 (3)	0.0511 (5)	
N2	0.46299 (14)	0.70215 (13)	0.1828 (3)	0.0550 (5)	
N3	0.32718 (12)	0.80332 (11)	0.1211 (2)	0.0397 (4)	
N4	0.26399 (12)	0.88834 (12)	0.0605 (2)	0.0403 (4)	
C1	0.28158 (17)	1.16575 (16)	0.0713 (3)	0.0534 (6)	
H1	0.3526	1.1746	0.1256	0.064*	
C2	0.21920 (19)	1.25088 (18)	0.0115 (4)	0.0626 (6)	
H2	0.2476	1.3166	0.0253	0.075*	
C3	0.11625 (18)	1.23664 (17)	−0.0673 (4)	0.0566 (6)	
C4	0.07182 (18)	1.14193 (19)	−0.0911 (4)	0.0625 (6)	
H4	0.0008	1.1346	−0.1464	0.075*	
C5	0.13374 (16)	1.05684 (17)	−0.0319 (3)	0.0536 (6)	
H5	0.1042	0.9916	−0.0479	0.064*	
C6	0.24016 (15)	1.06767 (15)	0.0515 (3)	0.0407 (5)	
C7	0.30545 (14)	0.97569 (14)	0.1164 (3)	0.0378 (4)	
C8	0.41148 (15)	0.98739 (15)	0.2543 (3)	0.0446 (5)	
H8A	0.4073	0.9702	0.3833	0.054*	
H8B	0.4330	1.0586	0.2552	0.054*	
C9	0.43346 (15)	0.79735 (15)	0.1685 (3)	0.0436 (5)	
C10	0.29301 (15)	0.70301 (14)	0.1038 (3)	0.0423 (5)	
C11	0.18178 (17)	0.67302 (16)	0.0591 (4)	0.0547 (6)	
H11A	0.1431	0.7122	−0.0514	0.066*	
H11B	0.1548	0.6907	0.1677	0.066*	
C12	0.1639 (2)	0.56042 (18)	0.0163 (4)	0.0671 (7)	
H12A	0.1819	0.5441	−0.1011	0.101*	
H12B	0.0920	0.5443	0.0024	0.101*	
H12C	0.2066	0.5210	0.1204	0.101*	

### Atomic displacement parameters (Å^2^)

**Table:**

	*U*^11^	*U*^22^	*U*^33^	*U*^12^	*U*^13^	*U*^23^
S1	0.0360 (3)	0.0450 (3)	0.0800 (4)	−0.0006 (2)	0.0126 (3)	0.0070 (3)
F1	0.0731 (10)	0.0583 (9)	0.1191 (13)	0.0317 (8)	0.0204 (9)	0.0218 (8)
N1	0.0518 (11)	0.0354 (9)	0.0640 (12)	0.0038 (8)	0.0113 (9)	−0.0002 (8)
N2	0.0464 (10)	0.0411 (10)	0.0745 (13)	0.0081 (8)	0.0108 (9)	0.0033 (9)
N3	0.0371 (8)	0.0311 (8)	0.0479 (9)	0.0031 (7)	0.0060 (7)	0.0004 (7)
N4	0.0357 (9)	0.0329 (9)	0.0489 (10)	0.0036 (7)	0.0053 (7)	−0.0006 (7)
C1	0.0437 (12)	0.0385 (12)	0.0739 (15)	0.0028 (9)	0.0084 (10)	0.0023 (10)
C2	0.0622 (15)	0.0361 (12)	0.0880 (18)	0.0041 (11)	0.0175 (13)	0.0021 (11)
C3	0.0581 (14)	0.0450 (13)	0.0682 (15)	0.0194 (11)	0.0194 (11)	0.0112 (11)
C4	0.0413 (12)	0.0615 (15)	0.0780 (16)	0.0107 (11)	0.0039 (11)	0.0074 (13)
C5	0.0426 (12)	0.0425 (12)	0.0702 (15)	0.0008 (9)	0.0049 (10)	0.0036 (10)
C6	0.0404 (11)	0.0367 (10)	0.0443 (11)	0.0030 (8)	0.0099 (9)	0.0008 (8)
C7	0.0364 (10)	0.0350 (10)	0.0414 (10)	−0.0004 (8)	0.0091 (8)	0.0005 (8)
C8	0.0394 (11)	0.0332 (10)	0.0550 (12)	−0.0019 (8)	0.0013 (9)	−0.0002 (9)
C9	0.0383 (10)	0.0400 (11)	0.0511 (12)	0.0050 (9)	0.0091 (9)	0.0035 (9)
C10	0.0483 (11)	0.0312 (10)	0.0446 (11)	0.0006 (9)	0.0071 (9)	−0.0015 (8)
C11	0.0498 (13)	0.0416 (12)	0.0682 (14)	−0.0055 (10)	0.0075 (11)	−0.0017 (10)
C12	0.0722 (17)	0.0486 (13)	0.0808 (17)	−0.0161 (12)	0.0210 (14)	−0.0076 (12)

### Geometric parameters (Å, º)

**Table:**

S1—C9	1.724 (2)	C4—C5	1.379 (3)
S1—C8	1.809 (2)	C4—H4	0.9300
F1—C3	1.359 (2)	C5—C6	1.393 (3)
N1—C10	1.294 (2)	C5—H5	0.9300
N1—N2	1.403 (2)	C6—C7	1.481 (3)
N2—C9	1.296 (2)	C7—C8	1.504 (3)
N3—C9	1.368 (3)	C8—H8A	0.9700
N3—C10	1.377 (2)	C8—H8B	0.9700
N3—N4	1.389 (2)	C10—C11	1.483 (3)
N4—C7	1.282 (2)	C11—C12	1.504 (3)
C1—C6	1.383 (3)	C11—H11A	0.9700
C1—C2	1.384 (3)	C11—H11B	0.9700
C1—H1	0.9300	C12—H12A	0.9600
C2—C3	1.351 (3)	C12—H12B	0.9600
C2—H2	0.9300	C12—H12C	0.9600
C3—C4	1.359 (3)		
			
C9—S1—C8	94.03 (9)	N4—C7—C8	123.28 (17)
C10—N1—N2	108.10 (16)	C6—C7—C8	119.81 (16)
C9—N2—N1	106.96 (17)	C7—C8—S1	112.80 (14)
C9—N3—C10	105.32 (16)	C7—C8—H8A	109.0
C9—N3—N4	128.67 (16)	S1—C8—H8A	109.0
C10—N3—N4	124.63 (15)	C7—C8—H8B	109.0
C7—N4—N3	115.66 (15)	S1—C8—H8B	109.0
C6—C1—C2	121.1 (2)	H8A—C8—H8B	107.8
C6—C1—H1	119.4	N2—C9—N3	110.27 (17)
C2—C1—H1	119.4	N2—C9—S1	128.82 (16)
C3—C2—C1	118.7 (2)	N3—C9—S1	120.86 (14)
C3—C2—H2	120.6	N1—C10—N3	109.33 (17)
C1—C2—H2	120.6	N1—C10—C11	126.78 (18)
C2—C3—C4	122.5 (2)	N3—C10—C11	123.81 (17)
C2—C3—F1	119.1 (2)	C10—C11—C12	113.32 (19)
C4—C3—F1	118.4 (2)	C10—C11—H11A	108.9
C3—C4—C5	119.0 (2)	C12—C11—H11A	108.9
C3—C4—H4	120.5	C10—C11—H11B	108.9
C5—C4—H4	120.5	C12—C11—H11B	108.9
C4—C5—C6	120.6 (2)	H11A—C11—H11B	107.7
C4—C5—H5	119.7	C11—C12—H12A	109.5
C6—C5—H5	119.7	C11—C12—H12B	109.5
C1—C6—C5	118.08 (19)	H12A—C12—H12B	109.5
C1—C6—C7	121.91 (18)	C11—C12—H12C	109.5
C5—C6—C7	120.01 (18)	H12A—C12—H12C	109.5
N4—C7—C6	116.73 (16)	H12B—C12—H12C	109.5
			
C10—N1—N2—C9	0.3 (2)	N4—C7—C8—S1	46.9 (2)
C9—N3—N4—C7	−26.4 (3)	C6—C7—C8—S1	−138.27 (16)
C10—N3—N4—C7	168.98 (18)	C9—S1—C8—C7	−49.59 (16)
C6—C1—C2—C3	−0.1 (4)	N1—N2—C9—N3	0.5 (2)
C1—C2—C3—C4	−0.4 (4)	N1—N2—C9—S1	−177.06 (16)
C1—C2—C3—F1	179.6 (2)	C10—N3—C9—N2	−1.0 (2)
C2—C3—C4—C5	0.3 (4)	N4—N3—C9—N2	−167.93 (18)
F1—C3—C4—C5	−179.7 (2)	C10—N3—C9—S1	176.72 (15)
C3—C4—C5—C6	0.2 (4)	N4—N3—C9—S1	9.8 (3)
C2—C1—C6—C5	0.6 (3)	C8—S1—C9—N2	−156.4 (2)
C2—C1—C6—C7	−179.4 (2)	C8—S1—C9—N3	26.27 (18)
C4—C5—C6—C1	−0.6 (3)	N2—N1—C10—N3	−1.0 (2)
C4—C5—C6—C7	179.3 (2)	N2—N1—C10—C11	−177.8 (2)
N3—N4—C7—C6	179.22 (16)	C9—N3—C10—N1	1.2 (2)
N3—N4—C7—C8	−5.8 (3)	N4—N3—C10—N1	168.80 (17)
C1—C6—C7—N4	−167.62 (19)	C9—N3—C10—C11	178.14 (19)
C5—C6—C7—N4	12.5 (3)	N4—N3—C10—C11	−14.3 (3)
C1—C6—C7—C8	17.2 (3)	N1—C10—C11—C12	−13.0 (3)
C5—C6—C7—C8	−162.73 (19)	N3—C10—C11—C12	170.6 (2)

### Hydrogen-bond geometry (Å, º)

**Table:**

*D*—H···*A*	*D*—H	H···*A*	*D*···*A*	*D*—H···*A*
C1—H1···N2^i^	0.93	2.51	3.428 (3)	172
C8—H8*A*···N1^ii^	0.97	2.50	3.410 (3)	156
C8—H8*B*···N2^i^	0.97	2.30	3.228 (3)	160

Symmetry codes: (i) −*x*+1, *y*+1/2, −*z*+1/2; (ii) *x*, −*y*+3/2, *z*+1/2.
